# Biological Mechanisms by Which Antiproliferative Actions of Resveratrol Are Minimized

**DOI:** 10.3390/nu9101046

**Published:** 2017-09-21

**Authors:** Yih Ho, Yu-Syuan Lin, Hsuan-Liang Liu, Ya-Jung Shih, Shin-Ying Lin, Ai Shih, Yu-Tang Chin, Yi-Ru Chen, Hung-Yun Lin, Paul J. Davis

**Affiliations:** 1 School of Pharmacy, College of Pharmacy, Pharmacy School, Taipei Medical University, Taipei 11031, Taiwan; yiho@tmu.edu.tw (Y.H.); oaykee@gmail.com (Y.-S.L.); 2 Taipei Cancer Center, Taipei Medical University, Taipei 11031, Taiwan; yajungshih@yahoo.com.tw (Y.-J.S.); cindylin812@yahoo.com.tw (S.-Y.L.); yutangchin@gmail.com (Y.-T.C.); 3 Department of Chemical Engineering and Biotechnology, Institute of Biochemical and Biomedical Engineering, National Taipei University of Technology, Taipei 10608, Taiwan; f10894@ntut.edu.tw; 4 Ph.D. Program for Cancer Biology and Drug Discovery, College of Medical Science and Technology, Taipei Medical University, Taipei 11031, Taiwan; 5 National Laboratory Animal Center, Taipei 11599, Taiwan; ashih@narlabs.org.tw; 6 Institute of Molecular Biology, Academia Sinica, Nankang, Taipei 11529, Taiwan; aquarlus9132@gmail.com; 7 Pharmaceutical Research Institute, Albany College of Pharmacy and Health Sciences, Rensselaer, NY 12208, USA; pdavis.ordwayst@gmail.com; 8 Department of Medicine, Albany Medical College, Albany, NY 12208, USA

**Keywords:** resveratrol, antiproliferation, cancer, tetrac, Nano-diamino-tetrac

## Abstract

Preclinical and clinical studies have offered evidence for protective effects of various polyphenol-rich foods against cardiovascular diseases, neurodegenerative diseases, and cancers. Resveratrol is among the most widely studied polyphenols. However, the preventive and treatment effectiveness of resveratrol in cancer remain controversial because of certain limitations in existing studies. For example, studies of the activity of resveratrol against cancer cell lines in vitro have often been conducted at concentrations in the low μM to mM range, whereas dietary resveratrol or resveratrol-containing wine rarely achieve nM concentrations in the clinic. While the mechanisms underlying the failure of resveratrol to inhibit cancer growth in the intact organism are not fully understood, the interference by thyroid hormones with the anticancer activity of resveratrol have been well documented in both in vitro and xenograft studies. Thus, endogenous thyroid hormones may explain the failure of anticancer actions of resveratrol in intact animals, or in the clinic. In this review, mechanisms involved in resveratrol-induced antiproliferation and effects of thyroid hormones on these mechanisms are discussed.

## 1. Introduction

Clinical evaluation of resveratrol and other well-studied polyphenols as anticancer agents have at best yielded variable results [1,2,3,4,5,6,7,8,9,10]. It is also important to point out that meta-analyses of clinical studies that have described the anticancer activity of polyphenols have not been based on prospective studies, but on a case–control study design, as noted by Grosso and co-workers [5].

The lack of congruence in preclinical and clinical studies of resveratrol and other polyphenols may reflect a low bioavailability for the administered compounds, a limited effectiveness of the metabolites, and the systemic toxicity that has served to limit dosing [2,3,11]. In addition, concentrations of the unconjugated form of resveratrol usually employed in in vitro studies are not obtained in vivo in tumor tissue [12] because of dose ceilings that are in place. Thus, much of the in vitro experience with resveratrol and other polyphenols is not relevant to clinical practice. Incomplete understanding of the mechanisms of the actions of resveratrol in cancer settings, in the intact animal or patient, may also be limiting our ability to translate preclinical results into clinical cancer management [2]. Bridging the translational gap between preclinical and clinical applications of resveratrol may be facilitated by monitoring circulating biomarkers of tissue action of resveratrol at various doses [13]. Such markers are insulin-like growth factor-1 (IGF-1) or IGF binding proteins.

In the current review, we describe certain anticancer functions of resveratrol, observed in vitro or in other preclinical studies, as they are reduced or blocked entirely by factors that come into play in the intact animal. We will point out that some of these anti-resveratrol actions are subject to modulation, with the result of restoring anticancer resveratrol effects.

## 2. Resveratrol Has Been Shown to Have Potential in Cancer Prevention and as an Antiproliferative Agent in Cancer

Resveratrol (3,4′,5-trihydroxystilbene), one of the best-studied stilbenes, exists principally in grapes, blueberries, peanuts, pistachios, and hops, and red wine also contains substantial amounts of resveratrol [14,15]. Resveratrol exists in both *cis*- and *trans*-stereoisomeric forms and it is the *trans*-isomer that is biologically active [15,16]. Preclinical studies have shown that resveratrol has cancer-preventive activity against various stages of development, for most cancers, including prostate, breast, stomach, colon, lung, thyroid, and pancreas [17]. Evidence also indicates that resveratrol is able to inhibit cancer cell proliferation and to induce cell cycle arrest and apoptosis in various types of cancer cells [18,19,20]. In addition, resveratrol induces differentiation in certain cell types [21,22,23].

The loss of mitochondrial membrane potential has been described as an early response to resveratrol that is relevant to apoptosis, and resveratrol treatment in isolated mitochondria has led to depolarization, suggesting that the drug may target mitochondria directly [24]. Resveratrol suppresses the activation of nuclear factor κB (NFκB) by upregulating mitogen-activated protein kinase (MAPK)-phosphatase-5 [25,26]; this serves to reduce the induction by NFκB of the expression of various anti-apoptosis genes [27]. The resveratrol-containing crude extract of seeds from Melinjo fruit (*Gnetum gnemon* L.) has been reported to induce apoptosis in cancer cells via caspase-3/7-dependent and caspase-independent mechanisms [28]. On the other hand, gnetin C, a resveratrol dimer, and active ingredient of seeds from Melinjo, can trigger both early- and late-stage apoptosis in cancer cells, at least in part by activating caspase 3/7-dependent mechanisms [28]. Resveratrol also induces the release of cytochrome c and Smac/Diablo from mitochondria, and subsequently, the activation of caspase-9 (4–8-fold) and caspase-3 (4–6-fold) after depolarization of the mitochondria [24].

The expression of matrix metalloproteinase (MMP) genes as a part of tumor invasion and metastasis is inhibited by resveratrol. The latter also decreases vascular endothelial growth factor (VEGF) levels in cancer cells, thus supporting anti-angiogenesis. Resveratrol has also been reported to sensitize cancer cells to ionizing radiation [29,30,31].

Prostaglandins are a product of cyclooxygenase (COX-2) action on arachidonic acid and have been shown to stimulate cell proliferation, promote angiogenesis, and suppress apoptosis—all of which support malignancy [32,33,34]. Resveratrol inhibits inflammation by directly blocking COX-2 activity. However, it was recently shown that resveratrol has another critical effect on COX-2, namely the induction of tumor cell antiproliferation via activated ERK1/2-dependent nuclear accumulation of COX-2 and activation/phosphorylation of p53-dependent apoptosis, in breast cancer, glioma, head and neck squamous cell cancer, and ovarian cancer cells [35,36,37,38].

**Figure 1:**
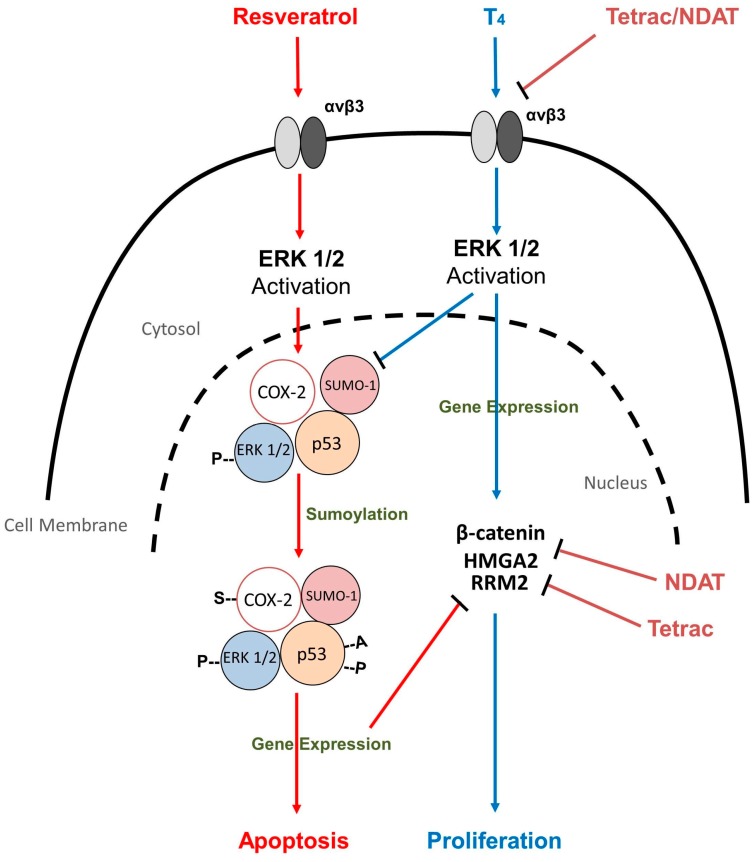
Thyroid hormone-induced interference with resveratrol-induced antiproliferation is blocked by tetrac and its nanoparticulate analog. Signal transduction pathways are involved in resveratrol-induced antiproliferation and thyroid hormone-mediated proliferation in cancer cells. Thyroid hormones, as T_4_, in physiological concentrations, stimulate cancer cell proliferation via the hormone receptor site on integrin αvβ3, on the cell surface. Activated ERK1/2 (pERK1/2) is required for hormone-dependent cell proliferation, as shown by the ERK1/2 cascade inhibition by the selective inhibitor, PD98059. A distinct receptor for the stilbene is also present on integrin αvβ3, through which resveratrol activates ERK1/2 and induces nuclear cyclooxygenase (COX-2) accumulation. Phosphorylated ERK1/2, induced by resveratrol, also translocates into the cell nucleus and complexes with inducible COX-2 in resveratrol-treated cancer cells. This is an essential upstream step in the induction by resveratrol of p53 phosphorylation at Ser-15, and consequent p53-dependent antiproliferation. Blocking resveratrol-induced nuclear accumulation of COX-2 inhibits p53 phosphorylation and antiproliferation. T_4_ inhibits the formation of the intranuclear pERK1/2-COX-2–p53 complex and consequent p53-dependent antiproliferation. The mechanism by which T_4_ inhibits the generation of pERK1/2–COX-2–p53 nuclear complexes in resveratrol-exposed cells is not yet known. Although the activation of ERK1/2 induced by hormones and resveratrol is additive, the competition for pools of ERK1/2 between thyroid hormones and resveratrol appears to divert kinases to the cell proliferation pathway, and may play an important role in the inhibition by T_4_ of resveratrol's pro-apoptotic action. Tetrac inhibits the expression of β-catenin and HMGA2, and NDAT inhibits the expression of RRM2, which is caused by resveratrol. In addition, both tetrac and NDAT activate the expression of Chibby, which binds to β-catenin and blocks its transcriptional activities. Thus, tetrac or NDAT may restore pro-apoptotic activity of resveratrol that has been lost due to the actions of endogenous thyroid hormones. Abbreviations: COX-2, cyclooxygenase-2; HMGA2, high mobility group AT-hook 2; tetrac/NDAT, tetraiodothyroacetic acid/nano–diamino–tetrac; RRM2, ribonucleoside diphosphate reductase subunit M2; Chibby, nuclear protein that directly binds to β-catenin and antagonizes its transcriptional activity; SUMO-2, small ubiquitin-related modifier-2.

The proposed mechanism for the antiproliferation induced by resveratrol is depicted in Figure 1. Via specific binding to the cancer cell surface integrin, αvβ3, resveratrol sequentially induces the activation of ERK1/2, nuclear localization of sumoylated COX-2, phosphorylation of p53, and antiproliferation. A specific COX-2 inhibitor, NS-398, is able to inhibit the complexing of nuclear COX-2 and activated ERK1/2, but does not affect resveratrol-induced ERK1/2 activation. Both ERK1/2 activation and nuclear COX-2 accumulation are required to complex with activated p53, to form a co-activator complex for p53-responsive genes, and for p53-dependent apoptosis in resveratrol-treated cells [39].

## 3. Inhibition by Resveratrol of Carcinogenesis in Animal Models

Resveratrol has limited bioavailability, but has been shown convincingly to prevent, and efficiently treat, tumors of the skin, esophagus, and gastrointestinal tract [40]. Different anticancer cellular mechanisms for resveratrol have been proposed, as noted above, and these include the induction of apoptosis and the inhibition of tumor-related angiogenesis [41,42]. Outcomes of such studies routinely reveal that the xenograft volumes and weights of resveratrol groups are less than those of control groups, with little or no systemic toxicity, (e.g., the net body mass of resveratrol groups in xenografted mice is not significantly different from that of control groups (*p* > 0.05) [43]).

At clinically achievable concentrations, the Melinjo (*Gnetum gnemon* L.) seed extract (MSE) and gnetin C mentioned above, significantly inhibit the proliferation of various cancer cells via apoptosis, without affecting normal cells. Gnetin C also has significantly greater antitumor activity than resveratrol [28]. Oral administration of MSE at 50 and 100 mg/kg per day significantly blocks tumor growth, induces intra-tumoral angiogenesis, and significantly reduces liver metastases in BALB/c mice bearing colon-26 tumors [28].

Resveratrol decreases tumor cell viability in vitro by 75% to 90%, primarily by the induction of apoptosis [24], and can inhibit tumor growth by more than 80% in vivo [24]. The apoptotic index of resveratrol-treated groups in animals is significantly higher than control groups. The expression of p53 and ERK activation are simultaneously and significantly increased in resveratrol-treated groups [43]. In NOD/SCID (non-obese diabetic/severe combined immune-deficient) mice xenografted with breast cancer stem-like cells (BCSC), resveratrol treatment (100 mg/kg/d) inhibited tumor growth. Analyses of the expression of autophagy-relevant LC3-II, Beclin1 and *Atg 7* genes in BCSCs indicated that resveratrol induced autophagy [44]. Resveratrol also suppressed the activity of the Wnt/β-catenin signaling pathway in BCSCs. Induction of an overexpression of β-catenin in BCSCs markedly decreased the cytotoxicity and autophagy caused by resveratrol in BCSCs [44].

### Disadvantages of Resveratrol

The pharmacokinetic and metabolite profiles of resveratrol have been defined in patients, e.g., those with colon cancer [12]. Resveratrol administered to healthy volunteers confirmed that it is well-tolerated and modulates enzyme systems involved in carcinogen activation and detoxification [45]. While it has promising anticancer potential, resveratrol has limited bioavailability, and thus efficacy is seen to be limited to tumors to which the agent can be directly applied, e.g., skin cancer and gastrointestinal tract tumors. It inhibits tumor growth effectively when it is administered intratumorally. The serum concentrations of resveratrol achieved in human subjects are in the low µM range (2–10 µM/L) [24]. Interestingly, there is no accumulation of resveratrol itself observed in tumor tissue [24], but metabolites, such as resveratrol glucuronide and piceatannol, are found in serum, liver, skin, and xenografted tumor tissue [46].

An optimal dose or dosing schedule for resveratrol has not been defined clinically. There is insufficient clinical evidence to validate a recommendation for the prophylactic administration of resveratrol [47]. For example, athymic mice received control diets or diets containing 110 μM or 263 μM of resveratrol, 2 weeks prior to a subcutaneous injection of melanoma cells. Resveratrol was rapidly metabolized, and at any concentration tested, resveratrol failed to inhibit human melanoma xenograft growth [46]. The higher levels of resveratrol tested (0.006% in food or 100 mg in slow-release pellets) tended, paradoxically, to stimulate tumor growth (*p* = 0.08–0.09) [44]. Piceatannol is a stilbenoid—a resveratrol-like compound—that does not affect the in vitro growth of a murine melanoma cell line, but stimulates the number of lung metastases significantly when these melanoma cells are directly injected into the tail veins of mice [46]. Thus, the effects of phytochemicals on cultured cells, and in the intact animal, may be inconsistent [46]. The foregoing information that is derived from a variety of tumor cells makes it difficult to devise and recommend additional clinical trials for resveratrol in solid tumors of any type [48].

Acute lymphoblastic leukemia (ALL) with translocation t(4;11) is a high-risk leukemia that is found in 60–85% of infants with ALL. It is often refractory to conventional chemotherapeutics after relapse. The efficacy of dietary resveratrol was examined in vivo in a model of the human t(4;11) ALL cell line SEM engrafted by tail vein injection into 5-week-old NOD.CB17-Prkdcscid/J mice, fed for 3 weeks with a regular diet or a diet containing 0.2% w/w resveratrol. Compared with the regular diet, dietary resveratrol did not postpone engraftment of the leukemia cells. Dietary resveratrol did not sensitize leukemic cells to vincristine, an antileukemic agent active in metaphase and an inducer of apoptosis [49].

## 4. Resveratrol-Induced Antiproliferation Is Opposed by Circulating Thyroid Hormone

Thyroid hormones play vital roles in normal physiological activities. However, studies have shown that thyroid hormones are able to interact with resveratrol when the latter is added in vitro or in vivo. Several interactions are listed in Table 1. The inhibition of cancer cells by thyroid hormones, through resveratrol-induced antiproliferation, has been studied in detail by our group. This action on cancer cells by thyroid hormones is mediated by a cell surface receptor for the hormone on integrin αvβ3 [50]. T_3_ (3,3′,5-Triiodo-l-thyronine) is responsible for the intracellular effects of thyroid hormones in normal cells, whereas l-thyroxine (T_4_)—usually viewed exclusively as a prohormone for T_3_—is the principal thyroid hormone ligand of the receptor on the plasma membrane of cancer cells. The physiological circulating concentration of free T_3_ is about 10^−11^ M and we have found this level of T_3_ to have no effect on resveratrol-induced antiproliferation. In contrast, circulating levels of free T_4_ do oppose the anticancer effects of resveratrol. The same cell surface protein (integrin αvβ3) has receptors for both thyroid hormones, and for resveratrol and other small molecules, but the binding sites are distinct from one another and do not interact on the integrin [51]. Agonist ligands of αvβ3 activate intracellular pools of MAPK (ERK1/2) and such activation has downstream (intracellular) consequences for thyroid hormones and resveratrol.

Thyroid hormones, particularly T_4_, play a key role in cancer progression [52,53,54,55,56,57]. In addition, thyroid hormones can overlap with the cellular functions of estrogen, enhancing cancer cell proliferation via nuclear estrogen receptor-α (ERα) Ser-118 phosphorylation in human ERα-positive cancer cells [56,58]. Thus, the signal transduction pathways of estrogen-stimulated ERα-positive cancer cell proliferation and that of thyroid hormones, are identical [58].

T_4_ inhibits the expression of antiproliferative BAD (Bcl-2-associated death promoter) and induces the expression of proliferative *BCL2* in malignant cells [59]. Thyroid hormones also control the expression of other *BCL2* gene family members, which have anti-apoptotic consequences. In addition, thyroid hormones decrease cellular abundances of caspases—such as caspase-3 and BAX, the gene product of which is pro-apoptotic in mitochondria [59]. Thus, the hormone is anti-apoptotic. Thyroid hormones can also prevent apoptosis damage in hypothyroid rat liver cells, induced by oxidative stress at the inner mitochondrial membrane [60]. The hormones induce the expression of myeloid cell leukemia 1 (MCL1), a principal Bcl-2-related protein that resides in the outer mitochondrial membrane, thus preventing mitochondrial membrane destabilization and the formation of channels by which the release of mitochondrial cytochrome c occurs, also leading to apoptosis [61]. T_4_ also increases the expression of *XIAP* (X-linked inhibitor of apoptosis) [59] and of HIF-1α [53,62]. Both have been shown to play an important role in anti-apoptotic functions. Chemotherapeutic agents with pro-apoptotic properties, such as paclitaxel, etoposide, and doxorubicin are subject to exportation from cells by the P-glycoprotein (P-gp, MDR1 or ABCB1) membrane *pump* [63]. The expression of the *MDR1* gene and the function of the *pump* gene product have been shown to be induced by thyroid hormones. Thyroid hormone-induced intracellular alkalization and Na^+^/H^+^ exchanger (NHE1) may also be related to the function of MDR1 [60,63]. Thus, thyroid hormones activate a panel of anti-apoptotic mechanisms.

These actions of thyroid hormones are rapidly initiated at the binding site for thyroid hormones on αvβ3. Thyroxine, at a physiological concentration, is able to interfere with resveratrol-induced antiproliferation by inhibiting serine phosphorylation of p53 and blocking resveratrol-induced cancer cell apoptosis [35]. The intracellular consequences downstream of the integrin-activated ERK1/2, for resveratrol at its receptor, and for thyroid hormones at their separate binding sites, are functionally opposed. Resveratrol induces nuclear COX-2 accumulation by an undefined mechanism, although resveratrol-induced sumoylation of COX-2 may be involved. In contrast, thyroxine prevents the accumulation of nuclear COX-2 in cancer cells and T_4_, in addition, blocks the formation of the nuclear complex of pERK1/2 and COX-2, which is induced by resveratrol in cancer cells [54]. RGD (Arg–Gly–Asp) peptide, an integrin αvβ3 antagonist, with its own recognition site, has been shown to inhibit certain functions of the integrin binding site for thyroid hormones and of the receptor for resveratrol [53].

Tetraiodothyroacetic acid (tetrac), the deaminated analog of T_4_, can inhibit thyroid hormone-induced activities by blocking the interaction of thyroid hormones with the cell surface integrin αvβ3 receptor. Tetrac does not affect resveratrol-induced biological activity [50,68], but thyroxine-induced inhibitory effects on the resveratrol-induced nuclear COX-2 accumulation, and later p53 phosphorylation, are prevented by tetrac [54]. How these signal transduction kinase steps are differentially regulated by T_4_ and resveratrol, is not yet clear. In addition to blocking the binding of thyroid hormones to integrin αvβ3, tetrac and a nanoparticulate derivative, nano–diamino–tetrac (NDAT), inhibit cancer cell proliferation [69] and block angiogenesis by disrupting the expression of a number of genes relevant to apoptosis and blood vessel formation. Studies of xenografts conducted in our lab have shown that the anticancer effect induced by low concentrations of resveratrol can be potentiated by both tetrac and NDAT. Tetrac inhibits the expression of the genes for *β-catenin* and *high mobility group AT-hook 2* (*HMGA2*), and thus potentiates resveratrol-induced antiproliferation in colorectal cancer cells. In addition, NDAT inhibits the expression of the ribonucleoside diphosphate reductase subunit *M2* (*RRM2*) gene, which is induced by resveratrol, but interferes with resveratrol-induced antiproliferation. The NDAT effect on *RRM2* thus facilitates resveratrol-induced antiproliferation. Both of these potentiating effects by tetrac and NDAT on resveratrol-induced anticancer growth are observed in xenografts of colorectal cancer HCT116 cells.

## 5. Conclusions

Resveratrol, via binding to a specific receptor on plasma membrane integrin αvβ3, induces ERK1/2- and p53-dependent antiproliferation in cancer cells. The induction of antiproliferation by resveratrol also depends upon the accumulation of resveratrol-induced nuclear COX-2, complexed with ERK1/2, and consequently with p53, to form a complex with transcriptional activity. Various endogenous circulating hormones affect cancer cell proliferation, metastasis, and invasion. The interactions between hormones and anticancer drugs have been intensively studied. We emphasize here that physiological concentrations of thyroid hormones, particularly T_4_, interfere with the well-described antiproliferative activity of resveratrol. Thyroid hormones and resveratrol bind to discrete receptors on plasma membrane integrin (αvβ3), to induce the activation of discrete intracellular pools of ERK1/2 in cancer cells, by a yet undefined mechanism. The ERK1/2 activated by thyroid hormones interestingly blocks ERK1/2-dependent formation of resveratrol-induced, p53-requiring nucleoprotein complexes; this action inhibits the antiproliferative effect of the polyphenol. In the xenografted animal model, and in the clinic, circulating physiological levels of thyroid hormones may be responsible for the suppression of resveratrol-induced anticancer activities in clinical studies. We suggest that the clinical efficacy of resveratrol in the treatment of cancer may be restored or potentiated by blocking the cancer cell surface receptor for thyroid hormone in vivo, or by reducing circulating levels of T_4_ and substituting T_3_ to maintain euthyroidism (euthyroid hypothyroxinemia) [70].

## Figures and Tables

**Table 1:** Studies that document interactions of thyroid hormones with resveratrol.

Study Design	Exposure/Result	Reference
Evaluation of resveratrol for its protective effects against fluoride-induced metabolic dysfunctions in the rat thyroid gland	Subacute exposure to sodium fluoride (dose of 20 mg/kg bw/day orally for 30 days) induced thyroidal dysfunction	[64]
Assessment of the effects of subcutaneous (s.c.) and oral administration of 17β-estradiol (E_2_) and the actions of resveratrol on the pituitary–thyroid axis in ovariectomized (OVX) female rats for 3 months	In vitro and in vivo studies demonstrated that serum resveratrol levels of 1.0 and 8.1 μM led to significant increases in total serum triiodthyronine (T_3_) levels. Ovariectomy induced thyroid stimuating hormone-β (TSHβ) mRNA expression in the adenohypohysis and E_2_ administration attenuated this effect. Treatment of OVX rats with s.c. E_2_ implants did not affect the pituitary–thyroid axis, whereas oral E_2_ benzoate (E_2_B) increased plasma TSH and total thyroxine (T_4_)	[65]
Assessment of the possibility that thyroid hormones are anti-apoptotic	In vitro, T_4_ induced ERK1/2 activation and caused minimal Ser-15 phosphorylation of p53. However, T_4_ did not affect the c-fos, c-jun and p21 mRNA abundances in proliferating human papillary and follicular thyroid cancer cells; cell proliferation was reduced by resveratrol co-incubation.	[55]
Examined the mechanism whereby T_4_ inhibits resveratrol-induced apoptosis in glioma cells	In vitro, T_4_ inhibited resveratrol-induced nuclear COX-2 and cytosolic pro-apoptotic protein (BcLx-s) accumulation. T_4_ inhibited resveratrol-induced apoptosis by interfering with the interaction of nuclear COX-2 and ERK1/2. T_4_ and resveratrol bind to discrete sites on integrin αvβ3.	[54]
Effects of treatment, with varying doses of resveratrol, on medullary thyroid cancer	In vitro, resveratrol treatment resulted in suppression of cell proliferation and increased cleavage of caspase-3 and poly(ADP-ribose)polymerase (PARP). A dose-dependent decrease in the abundance of ASCL1, a neuroedocrine transcription factor, was observed at protein and mRNA levels. CgA, a protein marker of hormone secretion, was also reduced. A dose-dependent induction of Notch2 mRNA was observed (qPCR).	[66]
Examination of the ability of polyphenol phytochemicals (including resveratrol) to induce redifferentiation in thyroid cancer cell lines.	The cell lines—TPC-1, FTC-133, NPA, FRO, and ARO—exhibited growth inhibition in response to resveratrol. Resveratrol decreased CD97 expression in FTC-133, NPA, and FRO thyroid cancer cell lines; there was increased expression of the differentiation marker, NIS, in FTC-133 cells, but no change in NPA, FRO, and ARO cells. Findings suggested that resveratrol may provide a useful therapeutic intervention in thyroid cancer redifferentiation therapy	[67]
